# New Treatment of Hydrocele

**Published:** 1841-10

**Authors:** 


*New Treatment of Hydrocele.* By M. Jobert.—This mode of treatment, which is founded on the same principles as that proposed by M. Velpeau for the cure of inguinal hernia, has already been put in practice by M. Jobert in several cases, and with every appearance of success. The following are the steps of the operation, as described in the report of the first case in which M. Jobert had followed the practice.

A small and very narrow bistoury was introduced at the middle and anterior part of the tumour, its cutting edge being directed inwards, and its back outwards. When the tunica vaginalis was pierced, M. Jobert depressed the handle of the bistoury, and carried it on in a direction parallel with the cord. Having reached with its point the summit of the tumour, he turned the cutting edge forwards as if to incise the integuments. This done, he withdrew the bistoury, dividing with its point the tunica vaginalis from the upper end of the sac to the point where the skin had been punctured. The bistoury was again immediately introduced by the same puncture, and the inferior part of the tunica vaginalis incised in the same manner. The fluid was then evacuated by the small puncture and compresses soaked in a solution of muriate of ammonia were applied. The patient suffered little during the operation, and nothing afterwards.

The day after the operation a small longitudinal depression was felt through the scrotum, corresponding with the point where the tunica vaginalis had been divided.

The operation was performed on the 22d of June, 1840, and the patient left the hospital about the middle of July, to all appearance cured.

In a case on which M. Jobert has since operated, in addition to the longitudinal incision he made likewise a transverse one, with the view of giving greater certainty of success.—*Edinburgh Monthly Journal of Medical Science.*

